# An Unusual Case of Bladder Wall Erosion Due to Prolonged Use of a Coudé Tip Urethral Catheter in a 79-Year-Old Man

**DOI:** 10.7759/cureus.95117

**Published:** 2025-10-21

**Authors:** Isteqal Miakhil, Steve Ndonga, Emmanuel Okpii, Sayed Iqbal Miakhil

**Affiliations:** 1 Surgery, Leicester University, Leicester, GBR; 2 Urology, Peterborough City Hospital, Peterborough, GBR; 3 Urology, North West Anglia NHS Foundation Trust, Peterborough, GBR

**Keywords:** bladder erosion, case report, catheter-related complication, coudé catheter, long-term catheterization, urology

## Abstract

We present the case of a 79-year-old man with long-term lower urinary tract obstruction managed with a coudé tip urethral catheter, who had an incidental finding of posterior bladder wall erosion diagnosed at cystoscopy for prostate enucleation. The posterior bladder wall erosion was treated conservatively. This rare but serious complication highlights the potential risk of prolonged use of rigid, curved-tip catheters and the need for careful catheter selection and regular reassessment in patients requiring long-term indwelling urinary drainage.

## Introduction

Coudé tip catheters are commonly used for difficult catheterizations, especially in male patients with enlarged prostates or urethral obstruction [1,2]. Coudé is a French word for elbow and refers to the curve of the tip [3]. In fact, it is the first catheter recommended in the American Urological Association’s algorithm for difficult catheterization, which states: "The angled tip facilitates passage through the prostatic urethra." Coudé tip has been shown to minimise risk of trauma, often reducing trauma rates from 3% to 0.2% of catheterizations [4]. Prolonged use of a rigid Coudé tip catheter can lead to localized trauma, pressure necrosis, and in rare cases, erosion of the bladder wall [4-6].

## Case presentation

A 79-year-old male with a known history of benign prostatic hyperplasia (BPH) and chronic urinary retention had been managed with an indwelling urethral catheter for over 12 months. Due to previous difficult catheterizations, a two-way Coudé tip catheter (16 Fr) was selected. The patient underwent catheter changes every 6-8 weeks without apparent complications.

**Figure 1:**
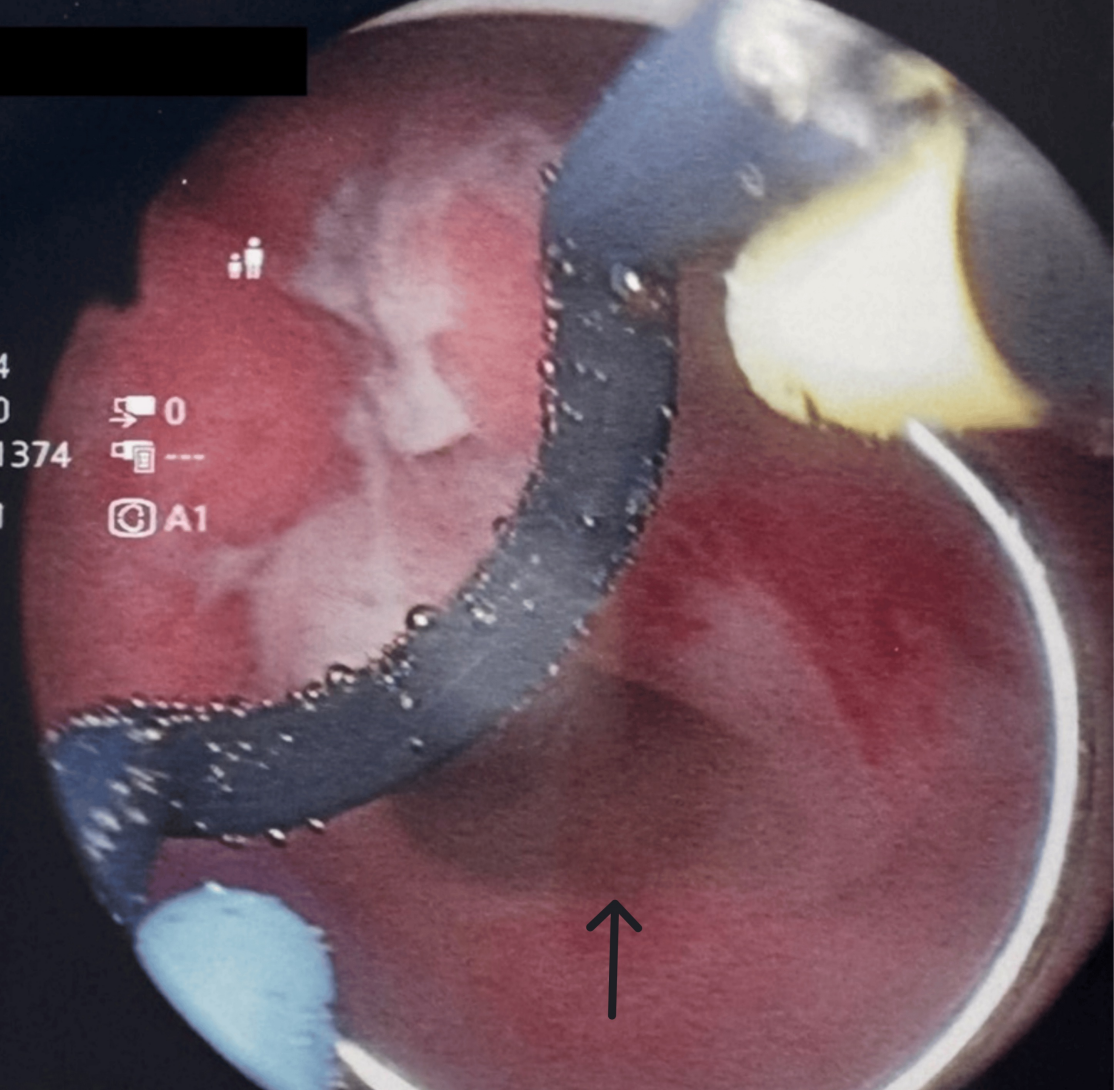
Routine cystoscopy with arrow head demonstrating posterior bladder wall erosion, most likely due to the coudé tip catheter.

**Figure 2:**
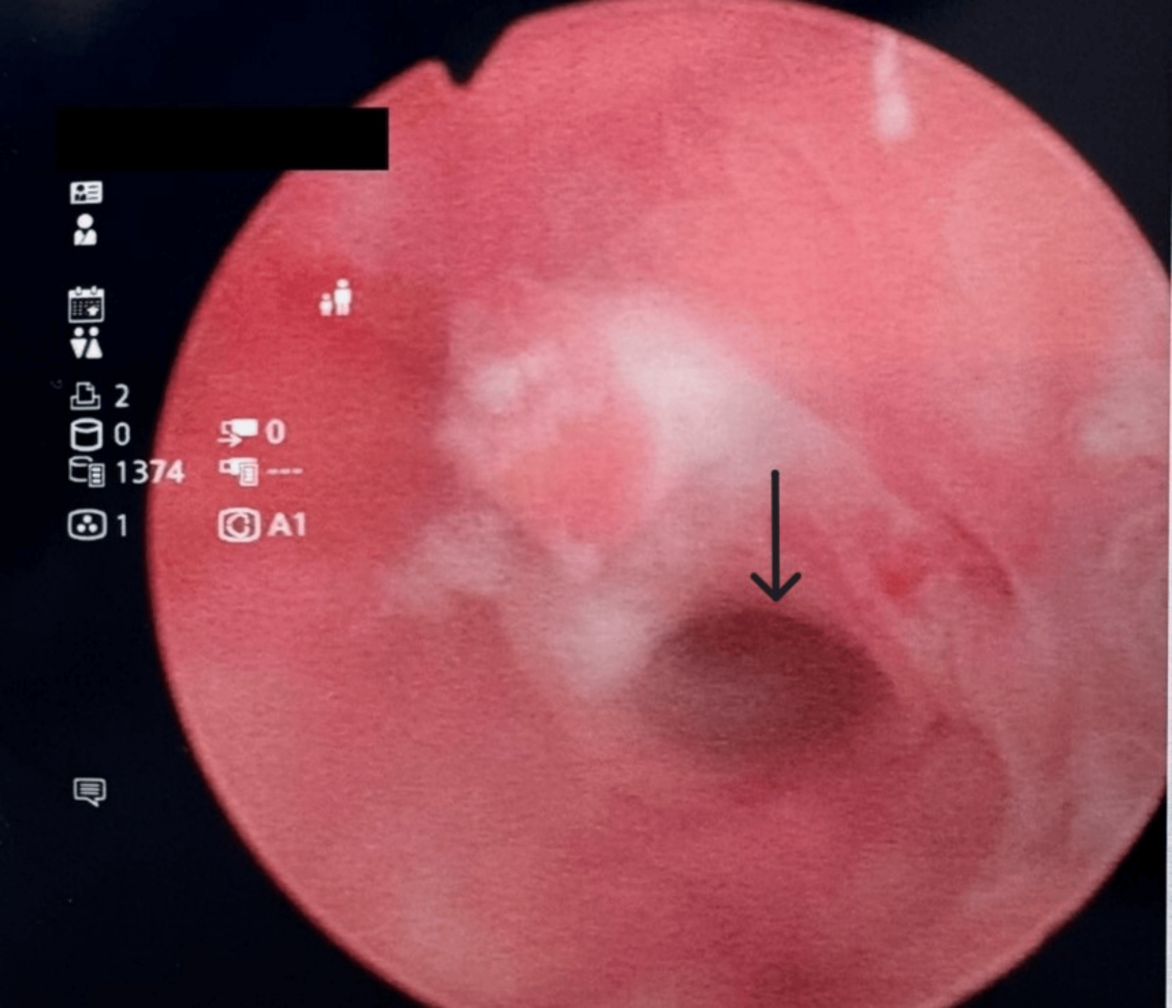
A close-up view of the posterior bladder wall erosion, demonstrated by the arrowhead.

**Figure 3:**
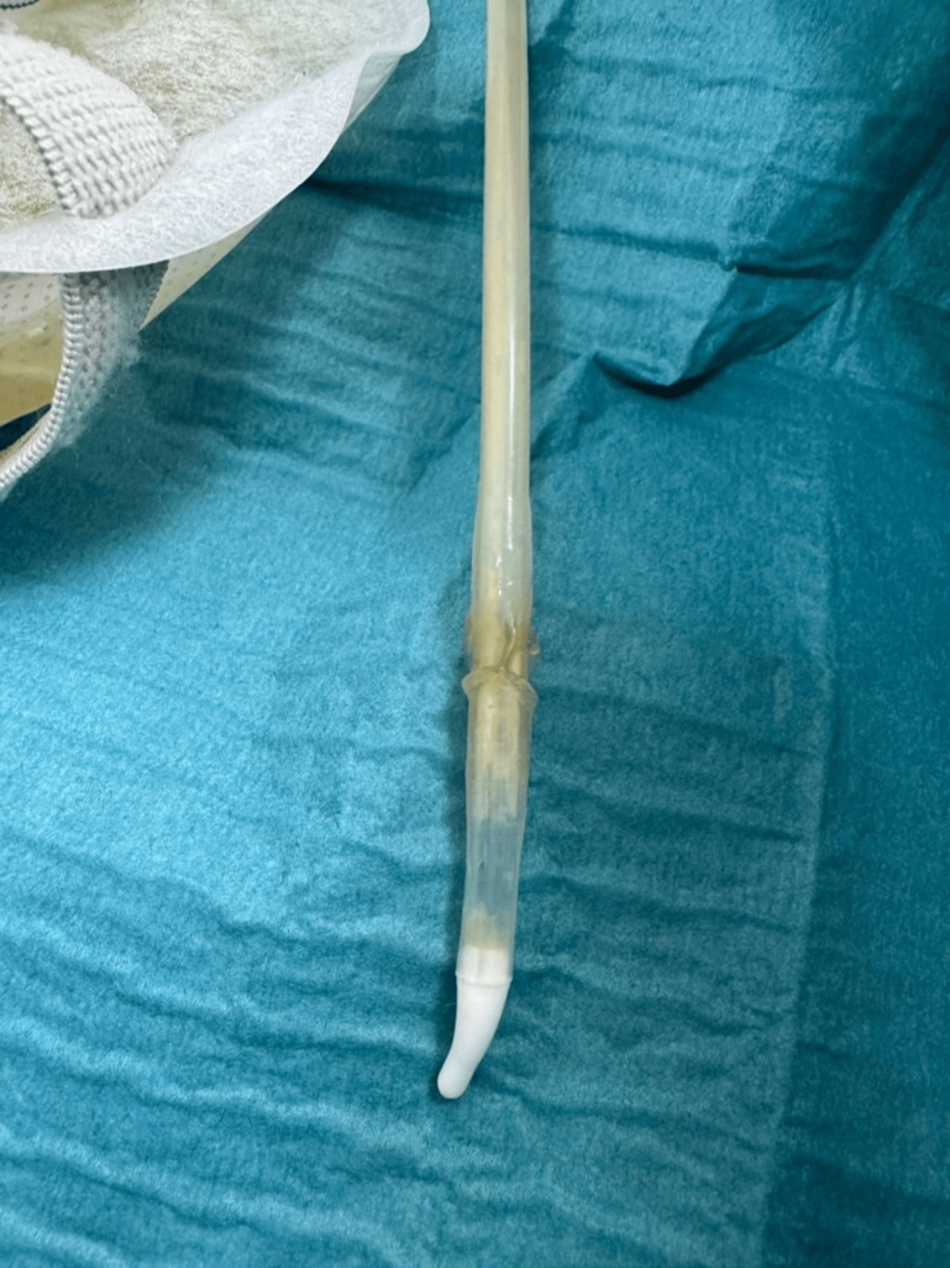
Coudé tip catheter removed prior to cystoscopy.

He presented for bipolar enucleation of his prostate to make him catheter-free. He did not have any specific complaints. A routine cystoscopy prior to surgery was performed, as seen in Figures 1-2, revealing significant erosion of the posterior bladder wall. While the location corresponded to the coudé tip position (see Figure 3), other potential causes, such as prior instrumentation or chronic inflammation, were considered less likely based on patient history and absence of other findings. There were no signs of peritonitis prior to surgery. 

The patient underwent bipolar enucleation of the prostate, and histology confirmed a benign diagnosis. He was reviewed in the clinic 12 weeks following surgery. To date, he is catheter-free and has had no urinary tract infections. The lower abdominal discomfort he previously experienced while the catheter was in place has resolved. He reports no haematuria and is satisfied with the outcome of the surgery. As he remained asymptomatic with no ongoing concerns, a cystogram was not deemed necessary.

## Discussion

Bladder erosion is a rare but life-threatening complication of indwelling urethral catheter use [5,6], and is more commonly associated with Foley catheters in cases of overinflated balloons or inadequate anchoring. Long-term catheterization can predispose patients to inflammatory changes within the bladder wall, further promoting the occurrence of perforation [5]. Literature describing coudé tip catheter usage and bladder wall erosion is very limited. Ogawa et al. described a case of intraperitoneal urinary bladder perforation due to an indwelling urethral catheter [7].

Coudé tip catheters are invaluable in bypassing prostatic obstruction [4]; however, their rigid, angled design can lead to pressure-related injury if left in situ for more than several weeks to months, as noted in published reports of catheter-related erosion. The tip may rest persistently against the bladder wall, leading to ischemia, mucosal erosion, and eventual perforation [4,5,7]. This effect is not limited just to the bladder, as any long-term indwelling urethral catheter can cause pressure necrosis of urethral tissue [1,4,5,8].

In our patient, there were no clinical signs of peritonitis, and bladder erosion was incidentally discovered during elective cystoscopy. Of note, our patient had a history of difficult catheterization and an indwelling coudé tip catheter, which was the most likely cause of the observed bladder wall erosion, although there was no evidence of full-thickness perforation.

Urinary bladder perforation can occur spontaneously in a weakened bladder wall [5-7], and prolonged coudé tip catheter use can contribute to this weakening by causing chronic pressure and localized ischemia.

Long-term indwelling urethral catheters can also cause pressure necrosis of the urethral tissue [1]. Coudé tip catheters can cause localized trauma, pressure necrosis, and in rare cases, erosion of the bladder wall.

We recommend some preventative measures for a rare but serious complication of coudé tip catheter usage. These include avoiding prolonged use unless clinically necessary and ensuring the tip is orientated properly (toward 12 o’clock) during insertion. A long-term indwelling catheter should only be used where there is no better alternative for the patient [1]. Other measures, such as the use of catheter valves, may promote bladder filling, storage, and micturition, which may help prevent loss of capacity and compliance, reducing the risk of a weakened bladder wall [1]. Good nursing care is also important in reducing indwelling catheter-related complications [1].

## Conclusions

This case underscores the need for vigilance in the selection and monitoring of indwelling catheters, particularly in elderly males with long-term urinary drainage needs. While coudé catheters serve a useful role, prolonged use of rigid catheters should be avoided; if necessary, patients require periodic cystoscopic monitoring. Clinicians should weigh the benefits of coudé catheters against potential risks in long-term use, and prioritize alternatives or timely definitive management whenever feasible.
